# 顶空固相微萃取-气相色谱-质谱法分析烟叶挥发性香味成分

**DOI:** 10.3724/SP.J.1123.2024.10004

**Published:** 2025-07-08

**Authors:** Xiaolong YAO, Yayun MA, Yuan LI, Gaojian SHI, Qianwei ZHOU, Yuhai ZHANG, Ningjie LUO, Lin ZHANG, Bin LI, Nan DENG, Lingjie MENG

**Affiliations:** 1.咸阳烟叶复烤有限责任公司，陕西 西安 712042; 1. Xianyang Tobacco Redrying Co. ，Ltd. ，Xi’an 712042，China; 2.西安交通大学大型仪器设备共享实验中心，陕西 西安 710049; 2. Core Facilities and Experiment Center，Xi’an Jiaotong University，Xi’an 710049，China; 3.中国烟草总公司郑州烟草研究院，河南 郑州 450001; 3. Zhengzhou Tobacco Research Institute of China National Tobacco Corporation，Zhengzhou 450001，China; 4.西安交通大学医学部，陕西 西安 710061; 4. Health Science Center，Xi’an Jiaotong University，Xi’an 710061，China

**Keywords:** 顶空固相微萃取, 气相色谱-质谱, 正交偏最小二乘-判别分析, 挥发性香味成分, 烟叶, headspace solid phase microextraction （HS-SPME）, gas chromatography-mass spectrometry （GC-MS）, orthogonal partial least squares-discriminant analysis （OPLS-DA）, volatile aroma components, tobacco

## Abstract

烟叶挥发性香味物质种类多且易受样品基质干扰，高效稳定的分析方法对于准确鉴定复杂基质中的挥发性香味成分至关重要。本文采用顶空固相微萃取-气相色谱-质谱联用技术分析了5个烟草产地中桔三（C3F）等级烟叶中挥发性香味成分。烟叶样品（1.0 g）在80 ℃条件下，使用80 μm二乙烯基苯/活性炭/聚二甲基硅氧烷（DVB/CWR/PDMS）萃取纤维顶空萃取30 min，在进样口处280 ℃解吸8 min。通过质谱数据库检索，初步检测到107种挥发性香味成分，采用内标法进行定量分析，酮类、芳香类和杂环类占70%以上（新植二烯除外）。结合正交偏最小二乘-判别分析（OPLS-DA）技术筛选出了代表性的差异物质。该方法及研究结果不仅为挥发性香味成分的标准化分析提供了参考，也为进一步深入挖掘与香味指标有关的物质，改善烟叶品质提供了理论依据。

## Abstract (en)

Volatile flavor compounds play vital roles when evaluating the flavor and quality of tobacco products. Pretreatment is always required owing to the wide range of flavor compounds and the complexity of the tobacco matrix. Solvent extraction （SE）， steam distillation （SD）， simultaneous distillation extraction （SDE）， supercritical fluid extraction （SFE）， and solid phase microextraction （SPME） are methods commonly used to extract and purify volatile flavor compounds. Among these methods， SPME coupled with headspace （HS） sampling has gained considerable attention in a variety of research fields because it combines sampling， extraction， concentration， and sample injection into a single procedure to deliver advantages that include convenient and simple sample preparation， small sample volumes， high sensitivities， and convenient operation.In this study， HS-SPME coupled with gas chromatography-mass spectrometry （GC-MS） was used to analyze the volatile aroma components in tobacco leaves. First， extraction efficiency was optimized by carefully evaluating multiple parameters， including types of solid phase microextraction fibers， extraction temperature， adsorption time， and desorption time. The number of chromatographic peaks， total chromatographic peak area， number of identified compounds， and internal-standard peak areas were used as indices. The optimized protocol involves incubating tobacco powder （1.0 g） under 80 ℃， extraction with an 80 μm divinylbenzene/carboxen/polydimethylsiloxane （DVB/CWR/PDMS） SPME needle for 30 min， followed by desorption from the fiber for a sufficient time （8 min）. These conditions led to a 1.6% RSD for the internal-standard peak area across five replicate experiments； hence， the developed method is highly repeatable. The volatile aroma components in tobacco leaves obtained from five different production areas were analyzed using the optimized parameters. A total of 107 volatile aroma compounds were identified， among which ketones， aromatics， and heterocyclic compounds accounted for more than 70% of the total volatile aroma components （excluding neophytadiene）. Tobacco leaves obtained from the Shangluo region contained the highest amount of total volatile aroma compounds， followed by leaves from Ankang， Hanzhong， Baoji， and Yan’an regions. Orthogonal partial least squares-discriminant analysis （OPLS-DA） was used to determine the main differential components from the various production regions， which identified 14 differential compounds （primarily ketones and alcohols）. This study provides a theoretical basis and reference for further exploring key volatile flavor compounds in leaves sourced from different production areas， as well as for identifying flavor-indicator substances and improving the quality of tobacco leaves.

食品风味物质是评价食品营养与质量品质的重要指标，目前风味物质中关于挥发性香味成分分析的研究有较多报道^［1-4］^。以烟草为例，烟草风味物质是卷烟产品风味和品质的重要影响因素，其中烟叶香味成分是烟草挥发性风味组分的主要组成部分。烟叶中的香味成分众多，组分复杂，含量较低。

由于烟叶中的风味物质种类多且基质复杂，在进行GC-MS分析前需要对烟叶样品预处理。目前，烟叶中风味物质的提取方法主要有溶剂萃取（SE）、水蒸气蒸馏（SD）、同时蒸馏萃取（SDE）、超临界流体萃取（SFE）和固相微萃取（SPME）等方法。其中，同时蒸馏萃取法是目前烟草行业内使用较多的一种香味成分提取方法，但该方法操作繁琐，耗时较长，溶剂消耗量大，在提取过程中可能会在糖类、氨基酸之间产生美拉德反应，使副产物增多，从而造成分析结果的重复性欠佳，不利于组分的定量分析。这些问题为该领域香味物质检测技术的标准化带来了很大挑战。

固相微萃取技术可实现样品采集与分析于一体，具有使用样品量少、灵敏度高、操作便捷等优势^［5］^。其中，顶空固相微萃取（HS-SPME）是将萃取纤维直接插入含有分析物的顶空样品瓶中，萃取纤维不直接接触分析物，通过纤维对样品瓶气相中的目标物进行萃取，因而受基质干扰较小，常与气相色谱-质谱（GC-MS）联用，在食品领域广泛用于复杂基质样品的分析^［6-11］^。顶空固相微萃取技术在烟草行业主要用于烟叶原料、烟气中挥发性物质的提取和检测^［12-16］^。陕西省环秦岭地区是我国重要的烟草种植区，但对该产地烟叶中香味成分的系统分析还未见报道。

本文以陕西省5个产地中桔三（C3F）等级烟叶为研究材料，通过优化HS-SPME-GC-MS的分析条件，建立了测定烟叶中挥发性香味成分种类与含量的方法，并结合正交偏最小二乘-判别分析（OPLS-DA）技术筛选出了不同产地烟叶的差异性物质。本文为烟草行业该领域技术方法的标准化发展提供了参考，同时也为烟叶原料物质基础特性研究及烟叶品质改善提供了技术方法与参考依据。

## 1 实验部分

### 1.1 仪器与试剂

电子分析天平（BSA224s， Sartorius，德国）、配有CTC PAL自动进样器的8890-7000D气相色谱-质谱联用仪、80 μm二乙烯基苯/活性炭/聚二甲基硅氧烷（DVB/CWR/PDMS）、65 μm二乙烯基苯/聚二甲基硅氧烷、100 μm聚二甲基硅氧烷、30 μm聚二甲基硅氧烷固相微萃取纤维针及顶空瓶（20 mL）购自美国Agilent公司。SPME萃取纤维在使用前均按照生产厂家所提供的条件进行活化处理。

乙酸苯乙酯（纯度98%）购自北京Solarbio公司；甲醇（色谱纯）购自德国Merck公司；正己烷（色谱纯）购自上海迈瑞尔生化科技有限公司。

样品为陕西省安康（AK）、宝鸡（BJ）、汉中（HZ）、商洛（SL）和延安（YA）产地烟叶，均为C3F 等级（咸阳烟叶复烤公司提供）。烟叶样品去除叶杆和叶脉后，切丝研磨成粉，采用40目筛网过滤，室温放置备用。内标物质乙酸苯乙酯采用正己烷溶解，配制质量浓度为100 μg/mL的内标溶液。

### 1.2 顶空固相微萃取条件

称取1.0 g样品置于20 mL顶空瓶中，常温放置于干燥器中，平衡24 h。将10 μL乙酸苯乙酯内标溶液加入上述顶空瓶中等待GC-MS分析，采用相同方式制备5份平行样品，分别检测分析。

固相微萃取纤维头：80 μm DVB/CWR/PDMS；萃取温度：80 ℃；萃取时间：30 min；解吸温度：280 ℃；解吸时间：8 min；老化时间：30 min。

### 1.3 GC-MS条件

HP-5 MS石英毛细管色谱柱（30 m×0.25 mm×0.25 μm，Agilent公司）；柱温程序设定：40 ℃保持2 min，以10 ℃/min的速率升温至290 ℃，保持5 min；载气为高纯氦气（纯度99.999%），载气流量1.0 mL/min；进样口温度为280 ℃，不分流进样。

离子源为电子轰击电离源（electron ionization，EI）；电子能量为 70 eV，离子源温度为230℃；四极杆温度150 ℃；接口温度 280 ℃；扫描范围*m/z* 35~500。

### 1.4 数据分析

采用MassHunter Workstation中的未知物分析软件进行色谱峰解卷积，利用未知物分析软件的Quant-My-Way功能编辑自动积分和解卷积方法，质谱数据采用美国国家标准与技术研究院（NIST 2.0）标准质谱库进行检索，结合手动检索分析，保留匹配分数不小于70分的挥发性物质。化合物的含量通过内标法定量。首先采用RStudio工具将检索结果进行整合，并进一步进行数据预处理和统计分析，获得包含样品信息、色谱峰保留时间、色谱峰面积及含量的列表。对于统计分析结果，使用Origin软件绘图，应用SIMCA进行OPLS-DA分析并计算重要性投影值（VIP）；使用TBtools软件绘制聚类热图。

## 2 结果与讨论

### 2.1 固相微萃取条件优化

以固相微萃取纤维的种类、萃取温度、萃取时间、解吸时间为考察因素，以上述汉中产地烟叶作为样品，通过色谱峰面积、色谱峰个数、鉴定化合物数量及内标峰面积的RSD等指标，综合优化评估样品分析采用的实验条件。

#### 2.1.1 SPME萃取纤维的影响

由于不同萃取纤维吸附的物质种类存在较大的区别，选择合适的萃取纤维尤为重要。本文测试了80 μm DVB/CWR/PDMS、65 μm DVB/PDMS、100 μm PDMS、30 μm PDMS 4种纤维。DVB/CWR/PDMS纤维表面是二乙烯基苯/活性炭/聚二甲基硅氧烷涂层，适用于挥发性和半挥发性（碳原子数为3~20）物质的提取，DVB/PDMS纤维适用于挥发性物质、胺类、硝基芳香类化合物的提取，100 μm PDMS纤维适用于极性挥发性化合物，30 μm PDMS纤维适用于非极性半挥发性化合物。从图1a中可以看出，DVB/CWR/PDMS组的色谱峰总面积和数量明显优于其他3种萃取纤维。从图1b看出，采用DVB/CWR/PDMS萃取纤维鉴定到的挥发性成分最多，同时能够保证内标峰面积的RSD较小（7.13%）。因此固相微萃取纤维选择DVB/CWR/PDMS。

**图1:**
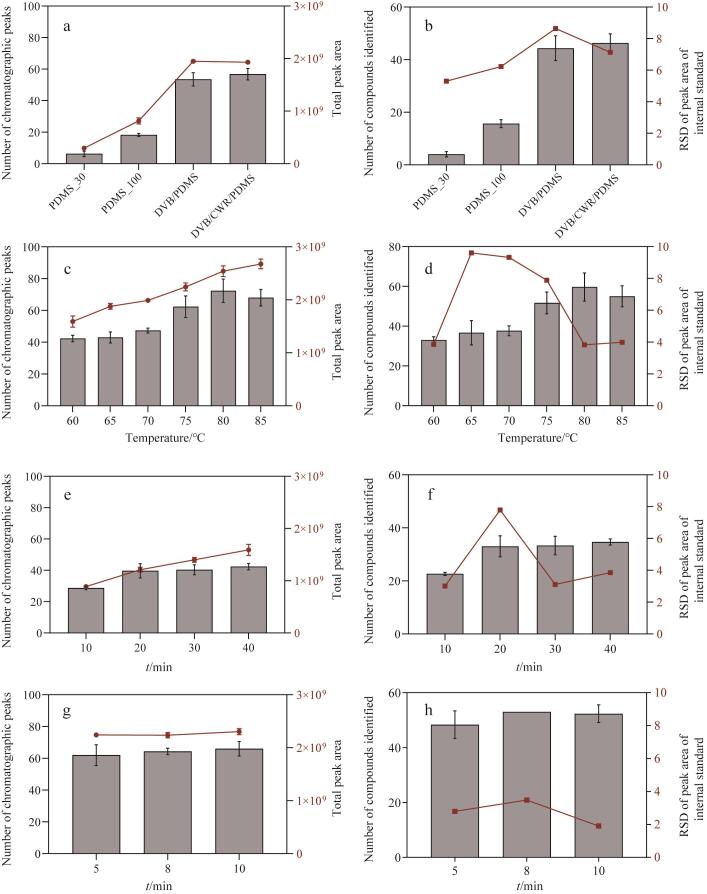
（a，b）固相微萃取纤维类型、（c，d）萃取温度、（e，f）萃取时间和（g，h）解吸时间对萃取效率的影响（*n*=3）

#### 2.1.2 萃取温度的影响

通过对样品进行加热，可以促进样品中化合物尤其是香味成分的挥发，提高萃取纤维对样品中挥发性化合物的吸附效率。从图1c可以看出，萃取温度为60~85 ℃时，色谱峰总面积和色谱峰数量随加热温度的升高而增加，当温度高于80℃后，待测物色谱峰个数和色谱峰总面积有所下降。同时从图1d也可以看出，当萃取温度为80℃，可以鉴定到的色谱峰数量最多，内标峰面积的RSD仅为3.83%，因此萃取温度选择80℃。

#### 2.1.3 萃取时间的影响

萃取时间与很多因素有关，如化合物的分配系数、扩散速率、样品基质、样品体积和萃取涂层厚度等。从图1e和图1f可以看出，随着萃取时间的延长，吸附量增加，色谱峰总面积和色谱峰数量增加明显，化合物的鉴定数量也增加，在20 min后基本趋于稳定。但是，内标峰面积的RSD在20 min条件下较高，为了提高结果的重复性，将萃取时间定为30 min。

#### 2.1.4 不同解吸时间的影响

萃取纤维提取化合物后，样品在进样口进行解吸附，延长解吸时间，有利于化合物解吸，但也可能会引入萃取纤维上的杂质，因此解吸时间不能过长。从图1g和图1h可以看出，当解吸时间为8 min时，挥发性化合物色谱峰数目和色谱峰面积最大，同时能够保证内标峰面积的RSD较小，仅为3.48%，因此解吸时间选择8 min。

### 2.2 重复性评价

为了考察HS-SPME前处理方法的重复性，制备5批次同条件的烟叶样品，采用上述优化条件，分别进行HS-SPME前处理和GC-MS分析。5批次样品重复分析的总离子流色谱图的堆叠图如图2所示，色谱峰基本完全重叠，5次平行分析中内标峰面积的RSD为1.6%，说明该方法的重复性良好。

**图2:**
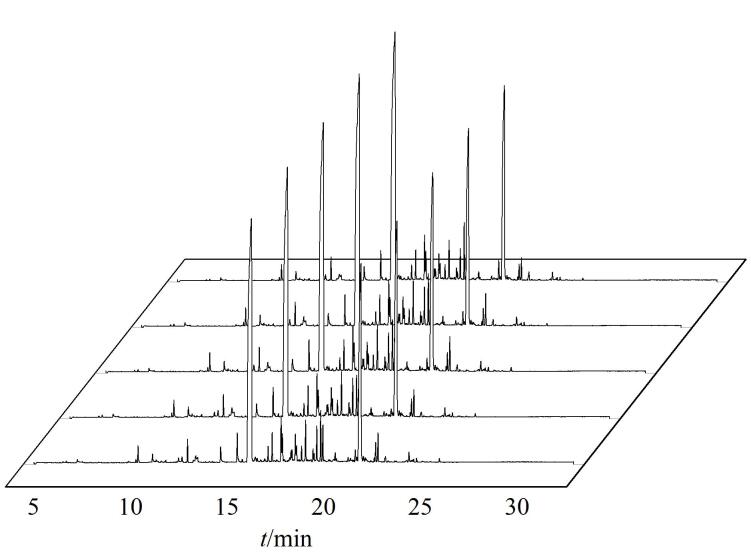
5批次烟叶样品的总离子流色谱图（*n*=5）

### 2.3 烟叶挥发性香味成分的总体情况

采用建立的HS-SPME-GC-MS方法对5个产地的样品进行分析，利用Masshunter workstation中的未知物分析软件对共流出组分进行解析。经谱图库检索与手动定性分析，从5个产地的烟叶中初步共检测到的挥发性香气成分如表1所示。去除不同产地烟叶中的含水率（安康15.25%、宝鸡15.99%、汉中15.78%、商洛15.89%、延安17.32%），以乙酸苯乙酯作为内标，计算挥发性香气成分含量，可以看出，香味成分主要包括酮类、杂环类、酯类、芳香类、醇类、醛类、酸类、烯烃类等。将所有挥发性香味成分的含量加和，获得挥发性香味成分总量，结果如表2所示。根据表2结果可以看出，商洛地区烟叶挥发性香味成分总量最高（41.90 μg/g），其次是安康和汉中地区，延安与宝鸡地区的含量较低，分别为32.46 μg/g和28.68 μg/g。这个结果与刘海轮等^［17］^在陕西烟叶品质特性研究中的结果以及王健强等^［18］^在陕西环秦岭烟叶生态与质量特色评价中的研究结果一致。在5个产地的烟叶中，含量最高的化合物均为新植二烯，在总香味成分化合物中的含量占比为42.77%~49.68%。其中，商洛产地烟叶中新植二烯含量最高，安康与汉中居中，宝鸡与延安最低，这可能与各产地的生态环境有关^［19］^。除新植二烯外，共检测到酮类32种，芳香类9种，杂环类11种，醇类218种，酯类25种，醛类10种，酸类2种。可以看出，各产地挥发性香气成分主要以中性与碱性为主，其中酮类、杂环类和酯类是相对含量较高的挥发性香气成分，占总量的70%以上（新植二烯除外）。整体趋势上，5个产地烟叶的含量变化趋势为酮类>酯类>杂环类>芳香类>醇类>醛类>酸类。从各类挥发性香气成分看，除新植二烯外，酮类含量最高，各产地平均可以占到总量（新植二烯除外）的35%以上，主要为2，3-二氢-3，5二羟基-6-甲基-4*H*-吡喃-4-酮、9-羟基-4，7-巨豆二烯-3-酮、6-乙基-5，6-二氢-2*H*-吡喃-2-酮、巨豆三烯酮等，大多为类胡萝卜素类降解产物，该类产物阈值低，刺激性小，香气质好，对烟叶香气贡献率大^［20］^。5个产地烟叶挥发性香味物质的种类数量如图3所示，可以看出，不同地区烟叶香味成分种类基本相似，均为酮类物质种类最多（20~25种），其次为酯类、醇类、芳香族类化合物，醛类和酸类香气物质数量较少。

**表1:** 5个产地烟叶中鉴定到的挥发性香味成分

Compound	Retention time/min	Ions （*m*/*z*）	Contents/（μg/g）				
			AK	BJ	HZ	SL	YA
Neophytadiene （新植二烯）	20.99	68， 82， 95	14.8	8.58	14.25	18.51	9.34
**Ketones （酮类）**							
Acetoin （3-羟基-2-丁酮）	4.43	4， 43， 27	0.04	/	/	/	0.01
1-（1-Cyclohexen-1-yl）ethanone （乙酰基环己烯）	7.56	81， 109， 43	/	0.01	/	/	/
6-Methyl-5-hepten-2-one （甲基庚烯酮）	9.36	43， 41， 69	0.10	0.14	0.09	0.10	0.06
1-（1*H*-pyrrol-2-yl）ethanone （2-乙酰吡咯）	10.65	94， 109， 66	0.13	0.11	0.10	0.09	0.2
3，4，5-Trimethyl-2-cyclopenten-1-one （3，4，5-三甲基-2-环戊烯-1-酮）	11.38	109， 81， 124	0.05	0.03	/	0.06	0.03
2-Acetyl-5-methylfuran （2-乙酰-5-甲基呋喃）	11.38	109， 124， 53	0.04	/	/	0.07	0.03
1-（3-Pyridinyl）ethanone （3-乙酰吡啶）	11.47	78， 106， 121	0.03	0.02	/	0.05	/
6-Methyl-2，3-dihydro-3，5-dihydroxy-4*H*-pyran-4-one （2，3-二氢-3，5二羟基-6-甲基-4*H*-吡喃-4-酮）	12.45	43， 44， 144	0.74	0.91	1.08	0.74	1.25
6-Ethyl-5，6-dihydro-2*H*-pyran-2-one （6-乙基-5，6-二氢-2*H*-吡喃-2-酮）	12.55	68， 97， 39	0.47	0.17	0.30	0.67	0.27
6-Propyl-5，6-dihydro-2*H*-pyran-2-one （6-丙基-5，6-二氢-2*H*-吡喃-2-酮）	12.55	68， 97， 79	0.47	/	0.23	/	0.04
2-Imidazolidinone （2-咪唑烷酮）	13.09	86， 30， 28	0.08	0.09	0.13	/	/
3-Ethyl-4-methyl-1*H*-pyrrole-2，5-dione （3-乙基-4-甲基吡咯-2，5-二酮）	13.66	139， 67， 53	0.17	0.11	/	0.25	0.12
*β*-Damascone （大马酮）	16.02	177， 192， 123	0.06	0.04	0.05	0.09	0.07
（*E*）-*β*-damascone （*β*-大马酮）	16.03	177， 69， 41	/	0.05	0.05	/	/
（*E*）-6，10-Dimethyl-5，9-undecadien-2-one （香叶基丙酮）	16.41	43， 69， 41	0.43	0.39	0.35	0.49	0.27
5-Hydroxy-3-methyl-1-indanone （5-羟基-3-甲基-1-茚酮）	17.38	147， 162， 119	0.32	0.28	0.32	0.38	0.32
Megastigmatrienone 1 （巨豆三烯酮1）	17.91	190， 148， 175	0.12	0.06	0.12	0.21	0.13
（*E*）-6，10-Dimethyl-5，9-undecatrien-2-one （假紫罗兰酮）	18.08	69， 41， 81	0.03	0.02	0.04	0.04	0.02
Megastigmatrienone 2 （巨豆三烯酮2）	18.12	190， 148， 175	0.36	0.19	0.42	0.66	0.37
4-Hydroxy-*β*-damascone （4-羟基-*β*-二氢大马酮）	18.52	69， 41， 121	0.14	0.10	0.19	0.20	0.12
Megastigmatrienone 3 （巨豆三烯酮3）	18.56	190， 148， 175	0.09	0.04	0.09	0.16	0.09
Megastigmatrienone 4 （巨豆三烯酮4）	18.70	190， 148， 175	0.34	0.16	0.43	0.62	0.35
Benzophenone （二苯甲酮）	18.77	105， 77， 182	0.03	0.02	0.03	0.05	0.03
3，5，5-Trimethyl-4-（3-hydroxy-1-butenyl）-2-cyclohexen-1-one （4-（3-羟基-丁烯基）-2-环己烯-1-酮）	18.91	108， 43， 152	0.81	0.35	0.61	2.52	0.66
3，5，5-Trimethyl-4-（3-hydroxybutyl）-2-cyclohexen-1-one （4-（3-羟基丁基）-3，5，5-三甲基环己-2-烯-1-酮）	19.59	135， 108， 93	0.03	0.01	0.02	0.07	0.03
*α*-Cyperone （*α*-香附酮）	19.73	218， 91， 147	0.10	/	/	0.19	0.07
Nootkatone （诺卡酮）	19.73	147， 121， 79	0.10	0.05	/	/	/
2，3，6-Trimethyl-1，4-naphthalenedione （2，3，6-三甲基萘醌）	20.33	200， 172， 118	0.12	0.06	0.13	0.17	0.07
Farnesylacetone （金合欢基丙酮）	21.76	69， 43， 81	0.19	0.11	/	0.23	/
7，9-Di-*tert*-butyl-1-oxaspiro （4，5）deca-6，9-diene-2，8-dione （7，9-二叔丁基-1-氧杂螺［^4^，^5^］癸-6，9-二烯-2，8-二酮）	21.88	57， 205， 55	0.44	0.25	0.44	0.56	0.52
**Aromatics （芳香类）**							
Methoxy-phenyl-oxime （甲氧基苯基肟）	9.01	39， 117， 105	/	/	/	/	0.06
5-Ethenyl-2-methoxyphenol （5-乙烯基-2-甲氧基苯酚）	14.86	135， 150， 77	0.40	0.41	0.28	0.23	0.29
6-Methylquinolin-8-amine （6-甲基-8-喹啉胺）	16.92	158， 157， 130	/	/	/	/	0.56
4-Ethenyl-2，6-dimethoxy-phenol （菜籽多酚）	17.88	180， 165， 137	/	0.03	/	/	0.05
Fluorene （芴）	18.29	166， 165， 167	0.05	0.03	0.04	/	/
2，2′，5，5′-Tetramethylbiphenyl （2，2′，5，5′-四甲基联苯）	19.33	195， 210， 180	/	0.03	0.05	/	0.03
Phenanthrene （菲）	20.61	178， 176， 76	0.22	/	/	/	0.09
9-Methylene-9*H*-fluorene （9-亚甲基-9*H*-芴）	20.61	178， 176， 179	0.24	0.07	0.15	/	0.10
Fluoranthene （荧蒽）	23.54	202， 203， 101	0.05	/	/	/	/
**Heterocyclics （杂环类）**							
1-Nitrosoazetidine （1-亚硝基氮杂环丁烷）	8.06	30， 41， 86	0.02	/	/	/	0.01
2-Propylfuran （2-丙基呋喃）	10.20	81， 53， 110	/	/	0.17	/	0.09
1，2，4，5-Tetrazine （1，2，4，5-四嗪）	10.21	28， 27， 82	/	0.02	/	/	/
Pyridazine （哒嗪）	10.91	80， 51， 52	0.07	0.12	0.12	0.09	0.05
Dihydrothiophene （二氢噻吩）	13.16	85， 86， 45	0.08	0.06	0.09	/	0.02
（*E*）-2-（1-Pentenyl）furan （（*E*）-2-（1-戊烯基）呋喃）	14.12	107， 79， 94	/	/	/	/	0.02
6-Amino-2，5-dimethyl-1*H*-indole （6-氨基-2，5-二甲基-1*H*-吲哚）	15.95	40， 159， 156	0.08	/	/	0.18	/
3-Methyl-1-phenyl-1*H*-pyrazole （3-甲基-1-苯基吡啶）	16.92	158， 77， 51	2.07	0.57	1.40	3.48	0.53
1-（1-Methyl-1*H*-pyrazol-4-yl）-3-piperidinamine （1-（1-甲基-1*H*-吡唑-4-基）-3-哌啶胺）	17.43	124， 137， 42	0.43	0.36	0.15	0.46	0.15
2，3′-Dipyridyl （2，3′-联吡啶）	17.60	156， 155， 130	1.44	0.73	2.05	3.62	0.88
Cotinine （吡啶吡咯酮）	19.66	98， 176， 118	0.12	0.05	0.13	0.31	0.07
3-Ethyl-3，4-dihydro-2（1*H*）-quinoxalinone （3-乙基-3，4-二氢-2（1*H*）-喹喔啉酮）	20.51	147， 119， 176	/	/	0.02	0.06	/
**Alcohols （醇类）**							
3-Methyl-1-butanol （异戊醇）	4.83	55， 42， 70	0.01	/	/	/	/
（2*S，*3*S*）-2，3-Butanediol （（2*S，*3*S* ）-（+）-2，3-丁二醇）	5.75	45， 43， 29	0.12	0.07	0.10	0.14	0.07
（2*R*，3*R*）-2，3-Butanediol （（2*R*，3*R*）-（-）-2，3-丁二醇）	5.75	45， 43， 27	/	/	0.10	/	0.08
4-Methanol-1*H*-imidazole （4-（羟甲基）咪唑）	6.97	97， 98， 69	0.04	/	/	0.05	0.04
Benzyl alcohol （苯甲醇）	10.22	79， 108， 107	0.60	0.42	0.20	0.68	0.35
（*R，R*）-1，2-Diphenylethanediol （（*R，R*）-（+）-氢化苯偶姻）	10.48	108， 107， 79	0.14	/	0.08	/	/
1-Phenylpropane-1，2-diol （1-苯丙烷-1，2-二醇）	10.54	108， 107， 79	0.15	/	0.13	/	/
1-Phenylpropane-1，2-diol （1-苯丙烷-1，2-二醇）	10.65	108， 107， 79	0.12	/	/	0.26	/
*α*-Methylbenzyl alcohol （苏合香醇）	11.56	107， 79， 77	0.42	0.27	0.16	0.57	0.32
2-Methyl-1-hexanol （2-甲基己-1-醇）	11.77	40， 57， 71	/	/	/	/	0.02
（*S*）-（+）-6-Methyl-1-octanol （（*S*）-（+）-6-甲基-1-辛醇）	11.94	55， 97， 70	/	0.03	/	/	0.02
4-Hydroxybenzyl alcohol （对羟基苯乙醇）	16.08	107， 138， 77	/	0.06	0.10	0.18	0.07
6-（3-Hydroxy-1-butenyl）-1，5，5-trimethyl-7-oxabicyclo［4.1.0］heptan-3-ol （6-（3-羟基-1-丁烯基）-1，5，5-三甲基-7-氧杂二环［4.1.0］庚烷-3-醇）	19.11	43， 125， 109	0.04	/	/	0.10	0.02
3-Methyl-1-butanol benzoate （苯甲酸异戊酯）	20.46	70， 105， 77	0.27	0.21	/	0.05	0.19
Geranyl linalool （香叶基芳樟醇）	21.76	69， 41， 81	/	/	/	0.32	/
Isocembrol （异瑟模环烯醇）	23.34	43， 81， 55	0.25	0.10	0.09	0.01	0.08
12-（1-Methylethyl）-1，5，9-trimethyl-4，8，13-cyclotetradecatriene-1，3-diol 12-（1-甲基乙基）-1，5，9-三甲基-4，8，13-环十四碳三烯-1，3-二醇	23.48	81， 43， 107	0.27	0.08	0.09	0.05	0.20
1-（2-Hydroxypropan-2-yl）-3a-methyl-6，10-dimethylidene-2，3，4，5，7，8，9，11，12，12a-decahydro-1*H*-cyclopenta［^11^］annulene-5，9-diol （1-（2-羟基丙-2-基）-3a-甲基-6，10-二甲基烯-2，3，4，5，7，8，9，11，12，12a-十氢-1*H*-环戊二烯［^11^］环烯-5，9-二醇）	23.48	95， 59， 107	0.04	/	/	/	0.18
**Esters （酯类）**							
Methyl valerate （戊酸甲酯）	6.41	74， 57， 85	0.02	/	0.03	/	/
Carbonic acid， monoamide， *N*-isobutyl-， benzyl ester （*N*-异丁基碳酸酰胺苯基酯）	10.41	91， 108， 65	/	0.13	/	/	/
Methyl 3-methylphenylcarbamate （甲氨基甲酸-3-甲苯酯）	11.37	108， 107， 77	/	/	/	0.13	0.09
Benzyl acetate （乙酸苄酯）	12.33	108， 91， 43	0.03	/	/	0.03	0.03
Methyl benzeneacetate （苯乙酸甲酯）	12.55	91， 150， 65	0.10	/	/	/	0.09
2-Chloroethyl benzoate （苯甲酸-2-氯乙酯）	13.96	105， 122， 77	0.10	0.04	/	0.19	0.11
4-Acetoxy-3-methoxystyrene （4-乙酰氧基-3-甲氧基苯乙烯）	14.86	150， 135， 43	0.42	0.42	/	/	/
3-Hydroxy-2，2-dimethylpropyl 3-hydroxypropanoate （2，2-二甲基-3-羟基丙酸新戊二醇酯）	16.00	73， 101， 88	/	0.02	0.10	/	/
Dimethyl phthalate （邻苯二甲酸二甲酯）	16.50	163， 77， 92	/	0.07	0.12	/	/
4-Nitrophenyl 2，5-difluorobenzoate （4-硝基苯基2，5-二氟苯甲酸酯）	17.05	40， 141， 153	0.12	0.06	/	/	/
Dihydroactinidiolide （二氢猕猴桃内酯）	17.67	111， 43， 137	0.58	0.42	0.54	0.64	0.41
2，2，4-Trimethyl-1，3-pentanediol diisobutyrate （2，2，4-三甲基-1，3-戊二醇二异丁酸酯）	18.24	71， 43， 159	0.07	0.05	0.10	0.06	0.07
Methyl myristate （肉豆蔻酸甲酯）	19.60	74， 87， 55	0.05	/	/	/	0.03
Undecyl benzoate （十一烷酸苯酯）	20.46	123， 105， 77	0.26	0.18	0.31	0.31	0.19
2-Methylbutyl benzoate （2-甲基丁基苯甲酸酯）	20.61	40， 105， 178	0.18	/	0.32	0.44	/
2，4-Dimethyl-3-pentyl benzoate （2，4-二甲基-3-戊基苯甲酸酯）	20.73	40， 429， 105	0.21	0.13	0.29	0.31	0.16
Isobutyl 4-octylphthalate （异丁基4-辛基邻苯二甲酸酯）	21.29	149， 40， 57	/	0.13	/	0.29	/
Isobutyl 4-heptylphthalate （异丁基4-庚基邻苯二甲酸酯）	21.30	149， 40， 57	/	0.14	0.34	/	0.15
Benzene-1，2-dicarboxylate （2-羟乙基苯甲酸酯）	21.49	105， 77， 123	/	0.07	/	/	/
2-Methylpropyl benzoate （苯甲酸异丁酯）	21.51	105， 123， 77	0.10	/	0.10	0.17	/
Heptyl benzoate （庚基苯甲酸酯）	21.51	123， 105， 77	/	0.06	0.12	0.15	/
（9*Z，*12*Z*）-3，7-Dimethyloct-6-en-1-yl octadeca-9，12-dienoate （（9*Z*，12*Z*）-3，7-二甲基辛-6-烯-1-基十八碳-9，12-二烯酸酯）	21.67	176， 40， 109	/	0.02	0.03	/	/
Methyl 10，13-dimethyltetradecanoate （10，13-二甲基十四烷酸酯）	21.75	74， 87， 57	0.27	/	/	/	/
Cetyl methyl ester （棕榈酸甲酯）	21.76	74， 87， 43	0.29	/	/	/	0.19
Butyl （*trans*-hex-3-enyl） phthalate （丁基（反式-己-3-烯基）邻苯二甲酸酯）	22.23	149， 82， 40	0.26	0.15	0.32	0.31	0.17
**Aldehydes （醛类）**							
3-Furaldehyde （3-糠醛）	6.59	95， 96， 39	/	0.06	/	/	0.11
Benzaldehyde （苯甲醛）	8.96	77， 106， 105	0.13	0.06	0.08	0.15	0.1
Benzeneacetaldehyde （2-苯基乙醛）	10.41	91， 120， 92	0.22	0.14	0.13	0.17	0.19
*N*-Methylpyrrole-2-carboxaldehyde （*N*-甲基-2-吡咯甲醛）	11.84	109， 108， 53	/	/	/	/	0.02
1，4-Benzenedicarboxaldehyde （对苯二甲醛）	12.56	134， 133， 105	/	/	/	/	0.01
2，6，6-Trimethyl-1，3-cyclohexadiene-1-carboxaldehyde （2，6，6-三甲基-1，3-环己二甲醛）	12.97	107， 91， 121	0.03	0.01	0.01	0.04	0.02
Vanillin （香草醛）	15.81	151， 152， 81	0.05	0.04	/	/	0.03
3-Ethylbenzaldehyde （3-乙基苯甲醛）	16.66	134， 133， 119	/	/	0.02	0.14	/
3，4-Dihydroquinoline-1-carboxaldehyde （3，4-二氢喹啉-1-甲醛）	17.53	132， 161， 118	/	0.10	0.25	/	0.13
5-［（*E*）-5-Hydroxy-3-methylpent-3-enyl］-1，4a-dimethyl-6-methylidene-3，4，5，7，8，8a-hexahydro-2H-naphthalene-1-carbaldehyde （5-［（*E*）-5-羟基-3-甲基戊-3-烯基］-1，4a-二甲基-6-亚甲基-3，4，5，7，8，8a-六氢萘-1-醛）	23.88	81， 107， 123	0.03	0.02	0.02	0.05	0.04
**Acids （酸类）**							
Nonanoic acid （正壬酸）	14.60	60， 73， 57	0.13	0.09	0.07	0.11	0.08
Benzeneacetic acid （苯乙酸）	14.66	91， 136， 92	0.18	0.11	0.21	0.17	0.29

AK： Ankang area； BJ： Baoji area； HZ： Hanzhong area； YA： Yan’an area.

**表2:** 不同产地各类挥发性香味成分含量

Category	Total number of aroma compounds	Contents/（μg/g）				
		AK	BJ	HZ	SL	YA
Neophytadiene	/	14.80	8.58	14.25	18.51	9.34
Ketones	31	6.30	3.89	5.38	8.71	5.24
Aromatics	9	2.80	1.07	1.76	3.70	1.61
Heterocyclics	12	2.24	1.35	2.73	4.71	1.30
Alcohols	18	2.45	1.25	1.04	2.41	1.63
Esters	25	3.09	2.08	2.72	3.04	1.68
Aldehydes	10	0.47	0.43	0.52	0.55	0.67
Acids	2	0.31	0.20	0.28	0.27	0.37
Sum	107	32.46	18.85	28.68	41.90	21.84

**图3:**
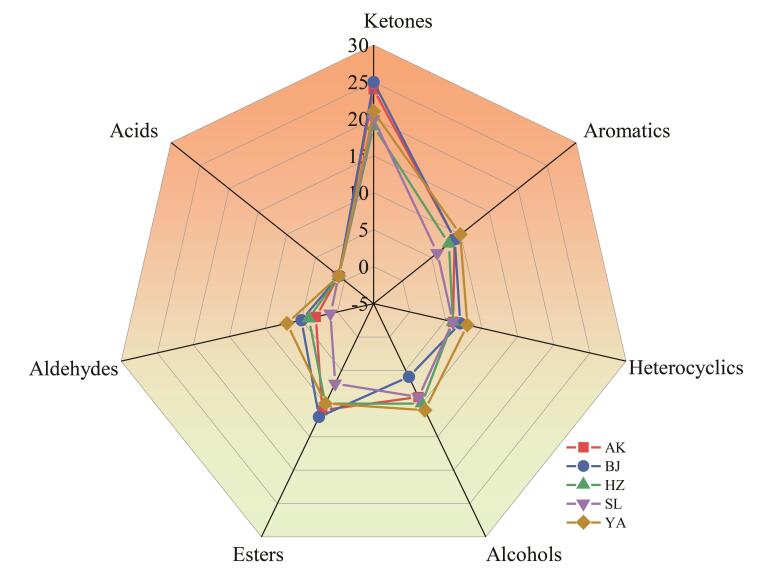
不同产地烟叶各类挥发性香气成分的数量

### 2.4 烟叶香味成分差异性分析

数据经标准化处理后，选择各产地共同鉴定的挥发性香味成分绘制含量热图，结果如图4所示。从图中可见，挥发性香味成分中含量较高的是新植二烯、2，3′-联吡啶、3-甲基-1-苯基吡啶、苯甲醇、二氢猕猴桃内酯。不同产地之间，商洛烟叶样品中新植二烯的含量最高，延安和宝鸡最低；各产地烟叶样品中2，3′-联吡啶、3-甲基-1-苯基吡啶与新植二烯的含量趋势相同；商洛与安康烟叶样品中苯甲醇含量最高，其次是宝鸡和延安，汉中最低；各产地烟叶样品中二氢猕猴桃内酯的含量比较接近（0.41**~**0.64 μg/g）。不同类别中酮类、杂环类、醇类、酯类、醛类等挥发性香气成分含量存在明显的差异，酮类中含量最高的是2，3-二氢-3，5二羟基-6-甲基-4*H*-吡喃-4-酮，杂环类化合物中含量最高的是2，3-联吡啶，醇类中苯甲醇含量最高，酯类中含量最高的是二氢猕猴桃内酯，醛类中含量最高的是辛醛，酸类中苯乙酸的含量最高。

**图4:**
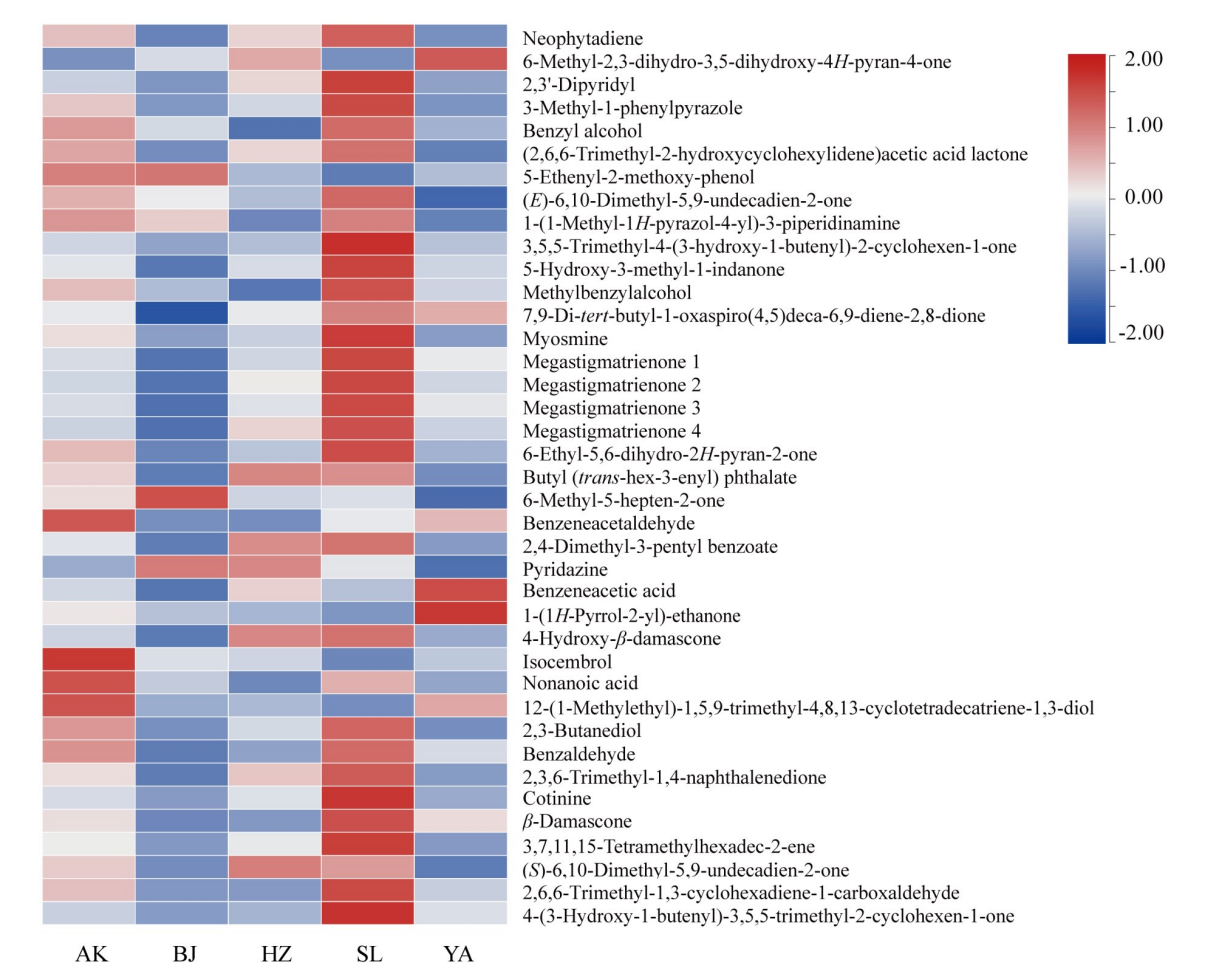
不同产地烟叶挥发性香味成分的含量热图

为进一步了解不同产地烟叶中挥发性香味成分的差异性，将其定量分析结果标准化处理后导入 SIMCA软件，采用OPLS-DA进行分析（图5），分析不同产地间的差异香味成分（图5a）。本次分析中的自变量拟合指数（*R* ^2^ *x*）为0.894，因变量拟合指数（*R* ^2^ *y*）为0.971，模型预测指数*Q* ^2^为0.949，说明模型匹配度较高，预测能力强。经过200次置换检验，*Q* ^2^回归线与纵轴的相交点<0，说明模型不存在过拟合（图5b），模型结果验证有效，可用于鉴别各产地香味成分差异性。VIP 值越大，表明该挥发性香味成分在不同产地之间差异越大（图5c）。因此以VIP>1为标准，结合方差分析结果（*P*<0.05）筛选出对模型分类贡献较大的变量有14种，包括5种酮类、4种醇类、2种杂环类、1种酯类、1种酸类、1种醛类。其中，酮类物质有甲基庚烯酮、6，10-二甲基-3，5，9-十一碳三烯-2-酮、4-羟基-*β*-二氢大马酮、6，10-二甲基-5，9-十一双烯-2-酮、*β*-二氢大马酮，主要为类胡萝卜素类降解产物。研究表明，*β*-二氢大马酮对香气量有正面影响作用，对香气质却起负面影响作用^［20］^。醇类物质中苯甲醇、苯乙醇为苯丙氨酸类降解产物，可使卷烟的烟气增加花香的香味^［21］^。根据模型可以看出，异瑟模环烯醇的VIP值最高，说明5个产地之间异瑟模环烯醇存在较大差异，该物质是中药海松子的主要成分，具有松子香、药香^［22］^。其次为苯乙酸，苯乙酸是一种对烟草香气和吸味有重要作用的挥发酸，可以降低烟气的碱性，减少刺激，使烟气变得醇和。

**图5:**
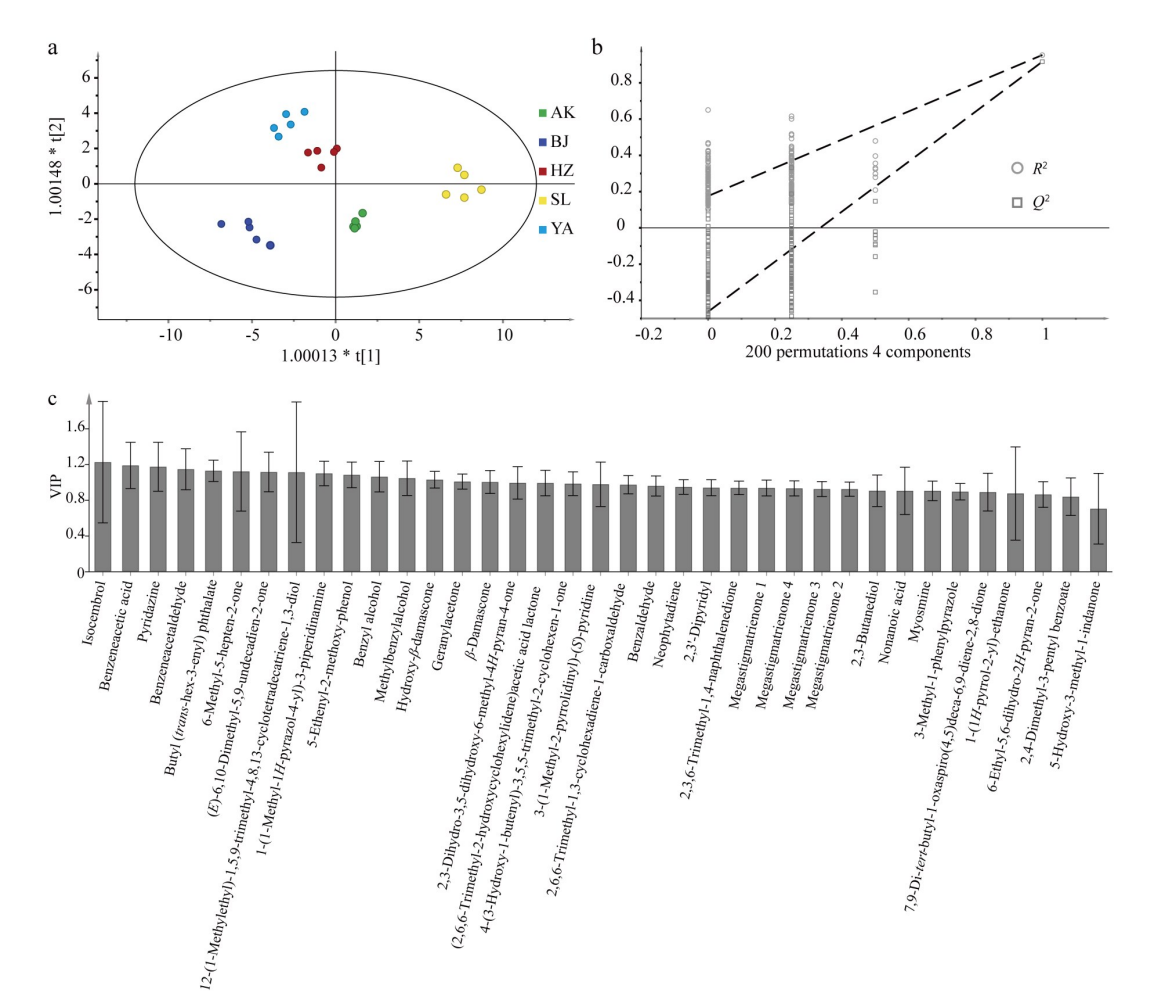
不同产地烟叶挥发性香味成分的OPLS-DA分析

## 3 结论

本文建立了一种基于HS-SPME-GC-MS的烟叶中挥发性香味物质的分析方法，优化了固相微萃取纤维类型、萃取温度、吸附时间和解吸时间等实验参数，并将其应用于不同烟草产地烟叶中挥发性香味成分的定性和定量分析。利用OPLS-DA筛选到不同产地间有显著差异的挥发性香味成分14种，其中较为突出的有异瑟模环烯醇、苯乙酸、甲基庚烯酮、苯甲醇、苯乙醇等，这些化合物可能与烟叶品质风格密切相关。与传统的溶剂提取法相比，该方法直接以烟叶粉末为样品，在减少样品用量的同时，避免采用大量的溶剂，省去了繁琐的离线样品前处理步骤，具有简单、快速、重复性好的优点。本方法作为传统方法的补充，有助于筛查烟叶中关键的风味物质基础，具有较好的普适性和应用前景。
